# G-protein coupled receptor, PI3K and Rho signaling pathways regulate the cascades of Tau and amyloid-β in Alzheimer’s disease

**DOI:** 10.1186/s43556-021-00036-1

**Published:** 2021-06-10

**Authors:** Smita Eknath Desale, Hariharakrishnan Chidambaram, Subashchandrabose Chinnathambi

**Affiliations:** 1 Neurobiology Group, Division of Biochemical Sciences, CSIR-National Chemical Laboratory (CSIR-NCL), Dr. Homi Bhabha Road, Pune, 411008 India; 2 Academy of Scientific and Innovative Research (AcSIR), Ghaziabad, 201002 India

**Keywords:** Alzheimer’s disease, Tau, PI3K/Akt, GPCR, TREM2, LPA, Rho GTPase

## Abstract

Alzheimer’s disease is a progressive neurodegenerative disease characterized by the presence of amyloid-β plaques in the extracellular environment and aggregates of Tau protein that forms neurofibrillary tangles (NFTs) in neuronal cells. Along with these pathological proteins, the disease shows neuroinflammation, neuronal death, impairment in the immune function of microglia and synaptic loss, which are mediated by several important signaling pathways. The PI3K/Akt-mediated survival-signaling pathway is activated by many receptors such as G-protein coupled receptors (GPCRs), triggering receptor expressed on myeloid cells 2 (TREM2), and lysophosphatidic acid (LPA) receptor. The signaling pathway not only increases the survival of neurons but also regulates inflammation, phagocytosis, cellular protection, Tau phosphorylation and Aβ secretion as well. In this review, we focused on receptors, which activate PI3K/Akt pathway and its potential to treat Alzheimer’s disease. Among several membrane receptors, GPCRs are the major drug targets for therapy, and GPCR signaling pathways are altered during Alzheimer’s disease. Several GPCRs are involved in the pathogenic progression, phosphorylation of Tau protein by activation of various cellular kinases and are involved in the amyloidogenic pathway of amyloid-β synthesis. Apart from various GPCR signaling pathways, GPCR regulating/ interacting proteins are involved in the pathogenesis of Alzheimer’s disease. These include several small GTPases, Ras homolog enriched in brain, GPCR associated sorting proteins, β-arrestins, etc., that play a critical role in disease progression and has been elaborated in this review.

## Introduction

Alzheimer’s disease (AD) is a progressive neurodegenerative disease that causes degeneration, neuronal loss and dementia [1]. Intracellular aggregates of Tau and extracellular amyloid-β plaques are the pathological hallmarks of AD [2, 3]. Amyloid precursor proteins (APP) are membrane proteins that are cleaved by β- and γ-secretases to form amyloid-β fragments under AD conditions. Tau is a microtubule-associated protein that binds tubulin and promotes microtubule stability [4, 5]. In AD brains, Tau is highly disordered and self-aggregates to form filaments. Several post-translational modifications of Tau are reported in AD brain that majorly includes phosphorylation, acetylation, glycation, glycosylation, methylation, sumoylation, etc., which limit its affinity to bind to microtubules [6–9]. In recent research days, Tau based therapy is attaining importance over amyloid-β and acetylcholine hypotheses [10–13]. Several drugs and molecules are being reported that inhibits Tau pathology, i.e., reversal of post-translational modifications, aggregation inhibition and disaggregation of pre-formed filaments, *etc*. [10–26]. In Alzheimer’s disease, several pathological signaling cascades are activated on exposure to these aggregated proteins which interacts with several membrane receptors of neuronal and glial cells. Signaling cascades mediated by various cell surface receptors in neurons as well as glial cells is a highly dynamic and interconnected system. Cellular processes such as phagocytosis, cell motility, immune function, metabolism and polarity are tightly regulated by cell-surface receptors and their signaling pathways [26–32]. Most of the cell surface receptors have an extracellular ligand-binding domain, a transmembrane region and an intracellular effector region, which is usually essential in transmitting the extracellular signal to the cell [33]. The intracellular transmission of signals is associated with kinase activities as a response to ligand binding.

One of which is receptor tyrosine kinase (RTK) that consists of intracellular tyrosine kinase domain irrespective of extracellular ligand-binding domain and a transmembrane domain [34]. Cytokine receptor type 1 and 2, multisubunit antigen receptors of B, T as well as macrophage cells comes under RTK family [35, 36]. Apart from RTKs, GPCRs are the largest family of surface receptors, which regulate physiological functions such as glucose and lipid metabolism, homeostasis, neurotransmission, immune responses, and other cellular functions [37]. GPCR consists of extracellular domain, a transmembrane region and intracellular domain that transmit the signal via various G-proteins (small GTPases). Gα and Gβγ subunits activate downstream effector pathways such as cAMP, protein kinase C (PKC), Ca^2+^ influx, PI3K, MAPK as well as ERK pathway [38]. In the downstream signaling cascades of receptors, protein kinase C (PKC) signaling acts as a feedback signaling for RTKs like insulin receptors, Met, Kit, EGFR, etc. Increase in intracellular Ca^2+^ levels by the production of diacylglycerol (DAG) via PLCγ is a well-known mechanism to activate PKC pathway [39]. The downstream signaling of GPCRs include activation of small GTPase Ras, PI3Ks, PKC, tyrosine kinase and arrestin, which follows ERK1/2 signaling pathway [40]. In Alzheimer’s disease, several GPCRs such as calcium sensing receptors, muscarinic acetyl choline receptors are activated by amyloid-β and Tau exposure and are involved in pathological signaling process that ultimately leads to neurodegeneration [41–44]. The different roles of GPCRs in amyloid-β synthesis, Tau phosphorylation, and Tau aggregation are clearly been reviewed [45–47]. Apart from the role of different GPCRs and their signaling through various G-protein dependent and independent pathways, there are various other GPCR regulating/ interacting proteins that take part in cellular signaling pathways for their growth and development. These proteins include small GTPases, Ras homolog enriched in brain (Rheb), GPCR associated sorting proteins (GASPs), receptor activity modifying proteins (RAMPs), *etc.* Among various small GTPases, the Ras superfamily of GTPases is well studied and are classified as five different sub-families: Ras, Rho, Rab, Ran, and Arf GTPases [48].

## PI3K/Akt signaling

The PI3K/Akt/mTOR is the central pathway for cell metabolism, migration, immune function,  and cell survival which is governed by many cell surface receptors. It involves formation of 3’- phosphorylated inositol phosphate, which activates Akt or mTOR ahead of PI3K activation. GPCRs activate PI3Kγ by regulating its catalytic subunit p110, the chemokine-induced cell migration of immune cells-mediated by this pathway [49]. In this study, we further discuss majorly the PI3K/Akt cell survival signaling and the other signaling partners associated with GPCRs.

### PI3K/Akt signaling-mediated by GPCRs

**Fig. 1:**
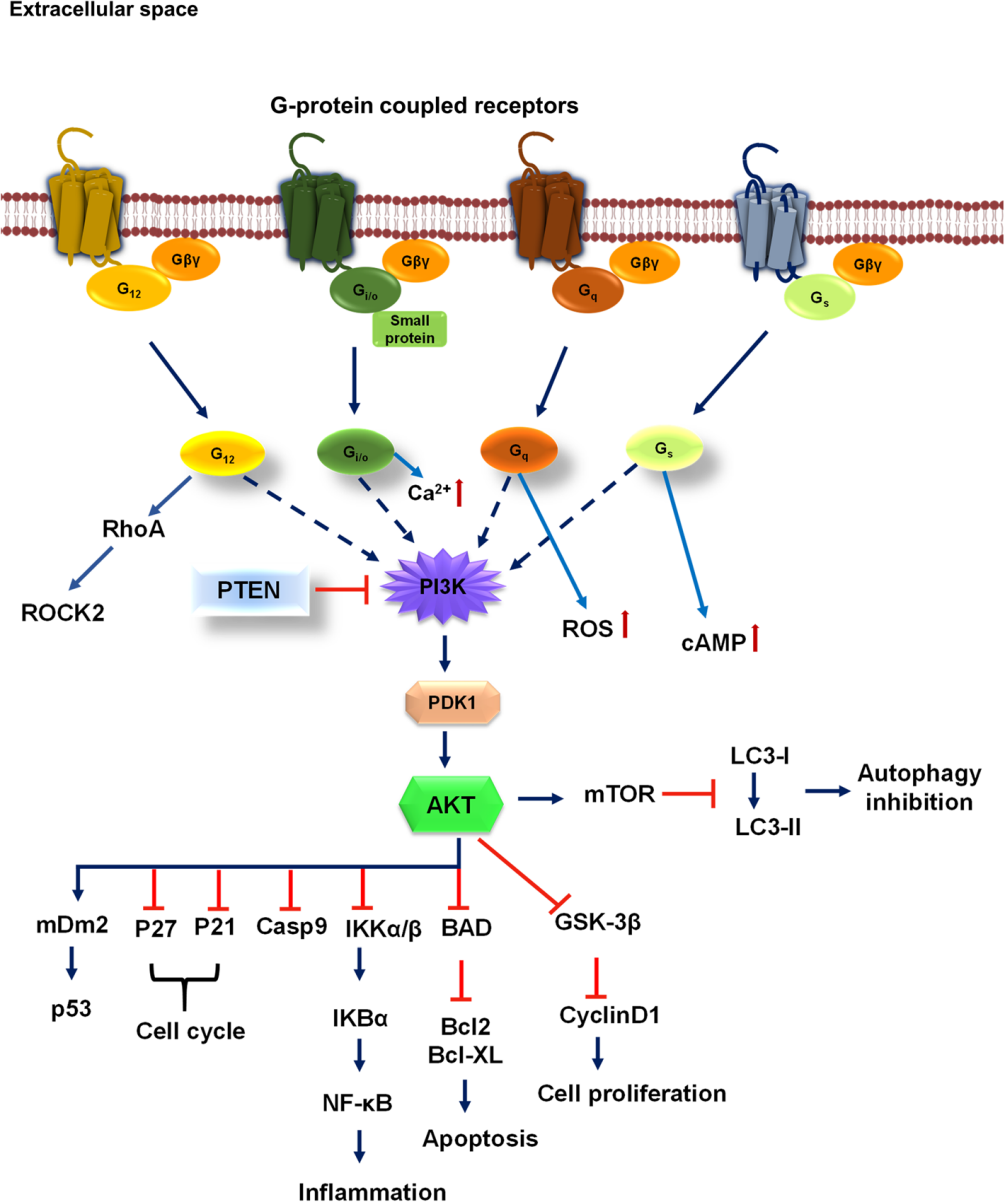
GPCR influence PI3K/Akt signaling. Among various GPCR G_12/13_, G_i/o_, G_q_, G_s_ are known to activate PI3K/Akt cell survival pathway via its Gα or Gβγ subunit. Along with PI3K/Akt signaling G_12/13_ activate RhoA/ROCK pathway, Gi/o involves in maintaining intracellular levels of Ca^2+,^ G_q_ with the ROS production, and Gas with secondary messenger cAMP. The regulation of PI3K is carried out by PTEN, a phosphatase of PI 3, 4, 5-P3, which inhibits PI3K directly. Upon activation of PI3K, Akt phosphorylation occurs *via* PDK1 kinase, followed by mTOR pathway activation, which inhibits autophagy response by inhibiting the conversion of LC3-l to LC3-ll. Akt specifically affects various downstream signaling pathway such as inhibition of GSK-3β, BAD, IKK, Casp9, P27, P21, and activates mDm2, which is involved in the p53-mediated response. This indicates a wide importance of Akt signaling in various cellular pathways

GPCRs are the type of integral membrane proteins, which transmit extracellular signals to intracellular space. These receptors form a superfamily of proteins, which bear a similar structural motif of seven transmembrane (TM) helices and are connected to three extracellular and intracellular loops [50]. The extracellular region of receptors act as an access point for ligands, and those include a range of molecules such as amines, lipids, proteins, peptides, nucleotides and even photons [51]. TM regions act as a structural core, which binds to ligands and transmits information to the intracellular space, *via* conformational changes. Intracellular region acts as a platform, which provides an interface for cytosolic signaling molecules. Despite of structural similarities, the signal-transducing mechanism varies as G protein-dependent and G protein-independent signaling pathways. The GPCRs significantly interact with G proteins while exerting their effector functions. G proteins that are coupled to GPCR include G_s_, G_q/11_, G_12/13_, and G_i_ [52, 53]. Stimulation of GPCR not only activates heterotrimeric G protein but also regulate intracellular effector signaling pathways such as activation of phospholipases, mitogen activated protein-kinases (MAPK), opening/closing of ion channels, and activation/inhibition of adenylyl and guanylyl cyclases [54, 55]. Various GPCRs are directly associated with small G-proteins and efficiently modulate downstream GPCR signaling network. GPCRs widely activate PI3K/Akt pathway *via* Gα and Gβγ, whereas Gβγ directly binds PI3K and activates the heterotrimeric protein consisting of either p110β or p110γ [55]. Along with authentic agonist, GPCR can recognize and respond to a wide array of ligands, which are known to activate PI3K/Akt signaling pathway. PI3K/Akt signaling is crucial since it regulates cell survival as well as metabolism [56]. Cells respond to a wide variety of stimuli *via* both GPCR and receptor-tyrosine kinases (RTK) under physiological conditions. Significant role of PI3K/Akt in cell proliferation, migration, apoptosis carried out by GPCR alone or in association with RTKs, is poorly recognized [57]. Human serotonin 5-HT receptor activates PI3K/Akt and MAPK signaling similar to VEGF via GPCR, which is important in angiogenesis [58]. Serotonin 5-HT receptor might activate dopaminergic neuron activity in a region-dependent manner *via* activating PI3K/Akt signaling through GPCR and extensively contributes to neurological functions. GPCR/PI3K/Akt signaling cascade is believed to be an important therapeutic target to understand neurodegenerative diseases [56]. Several medicinal herbs have been studied to initiate neuroprotective effects *via* PI3K/Akt pathway that include curcumin, danshensu, puerarin, etc [56]. Docosahexaenoic acid (DHA), a polyunsaturated fatty acid directly influences the oligomerization of GPCR by changing the cell membrane composition. GPCR signaling is modulated by DHA and it is observed to increase neuronal survival by phosphorylating Akt [59, 60]. Ratios of n-6/n-3 polyunsaturated fatty acids (PUFA) significantly influence PI3K/Akt signaling and inhibits inflammation [61]. The free fatty acids can increase glucose uptake by cell *via* PI3K/Akt signaling [62]. On activation, PI3K generates secondary lipid mediator phosphatidylinositol 3,4,5- triphosphate (PIP3), which phosphorylates Thr308 on Akt *via* phosphoinositide-dependent kinase 1 and at Ser473 *via* rapamycin complex 2. Dual phosphorylation of Akt is necessary for complete activation, however Thr308 is indicative of PI3K activity [63, 64]. Up-to date, several GPCR activating ligands have been identified such as sphingosine 1-phosphate, lysophosphatidic acid, stromal cell-derived factor, prostaglandin E2, carbachol, isoproterenol, thyroid-stimulating hormone (TSH), follicle stimulating hormone (FSH), and luteinizing hormone (LH)/choriogonadotropin (CG), *etc.* [57]. Activation of PI3K signaling pathway is majorly carried out in response to extracellular signal which is mediated by cell-surface receptors such as GPCRs, RTKs, integrin, growth factor receptors, where Akt plays a crucial role in activating this pathway [65]. The receptor-mediated activation of PI3K results in the production of phosphatidylinositol 3,4,5- triphosphate (PIP3) from phosphatidylinositol 4,5 bisphosphate (PIP2). PIP3 can be reversed to PIP2 by the enzyme, phosphatase and tensin homolog (PTEN) and SH-domain-containing inositol polyphosphate 5-phosphatase (SHIP). Both Akt and phosphoinositide-dependant kinase (PDK) are enriched at the plasma membrane *via* PIP3 through their pleckstrin homology domain [66] (Fig. 1).

PI3K/Akt/GSK-3β pathway plays a crucial role in Alzheimer’s disease (AD), as it is one of the leading causes of Tau hyperphosphorylation. Glycogen synthase kinase-3β (GSK-3β) regulates the neuronal stress response via CREB, which regulates BDNF an important neuropeptide for memory retention, synaptic plasticity, *etc*. [55, 67]. Tau hyperphosphorylation and aggregation is generally led by several kinases, which include GSK3β, CDK-5, p38, p38-MAPK *etc.* PI3K/Akt pathway impairment majorly deals with the increased activity of GSK-3β and hence Tau aggregation, which ultimately forms neurofibrillary tangles (NFTs) [68, 69]. Aβ-induced hyperphosphorylation of Tau is mediated through defective Akt/GSK-3β pathway, which provides a therapeutic link [70]. Anomalously, downregulation of Akt and subsequent elevated GSK-3β activity is related to brain dysfunctions, since Akt is a key regulator of cell survival and apoptosis [68]. Beside several signaling pathways, GPCRs are found to bind to β- and γ-secretase, the key enzymes in amyloid protein precursor (APP) processing and amyloid plaque deposition [46]. In the GPCR superfamily, M1mAChR, A_2A_ receptor, and δ-opioid receptor are known to influence BACE1 activity, which is important in Aβ production. Along with receptors, GPCR-associated sorting proteins (GASPs), small G proteins such as Rabs, ADP-ribosylation factor 6 (ARF6) mediate BACE1 activity [47]. Muscarinic acetylcholine receptor (mAChR), especially M1 and M3 are found to increase soluble APP *via* α-secretase activity and reduces Aβ production. The release of sAPPα is assumed to be *via* PKC activation or interaction with calcium released *via* IP3 pool and DAG [71]. The opioid system, which is involved in neuroendocrine function as well as immune response is associated with abnormal Aβ production upon disrupted signaling. The enhancement of BACE1 and secretases activity are also regulated by opioid signaling of delta-opioid receptor (DOR) and naltrindole (NTI) [72, 73]. Adenosine receptor present on glia and neurons function in synaptic transmission and neuronal excitability. Adenosine A1 receptor found to be involved in Tau phosphorylation and also in Aβ production. These receptors are found to be colocalized with NFTs and amyloid plaques in the AD brain [45, 74]. Along with GPCRs, small GTPases such as Ras, Rac/cdc42/Rho, Rab, Sar1/ARF and Ran family have a significant role in AD pathogenesis [75] (Fig. 1).

### PI3K/Akt survival signaling pathway by TREM2 receptor

**Fig. 2:**
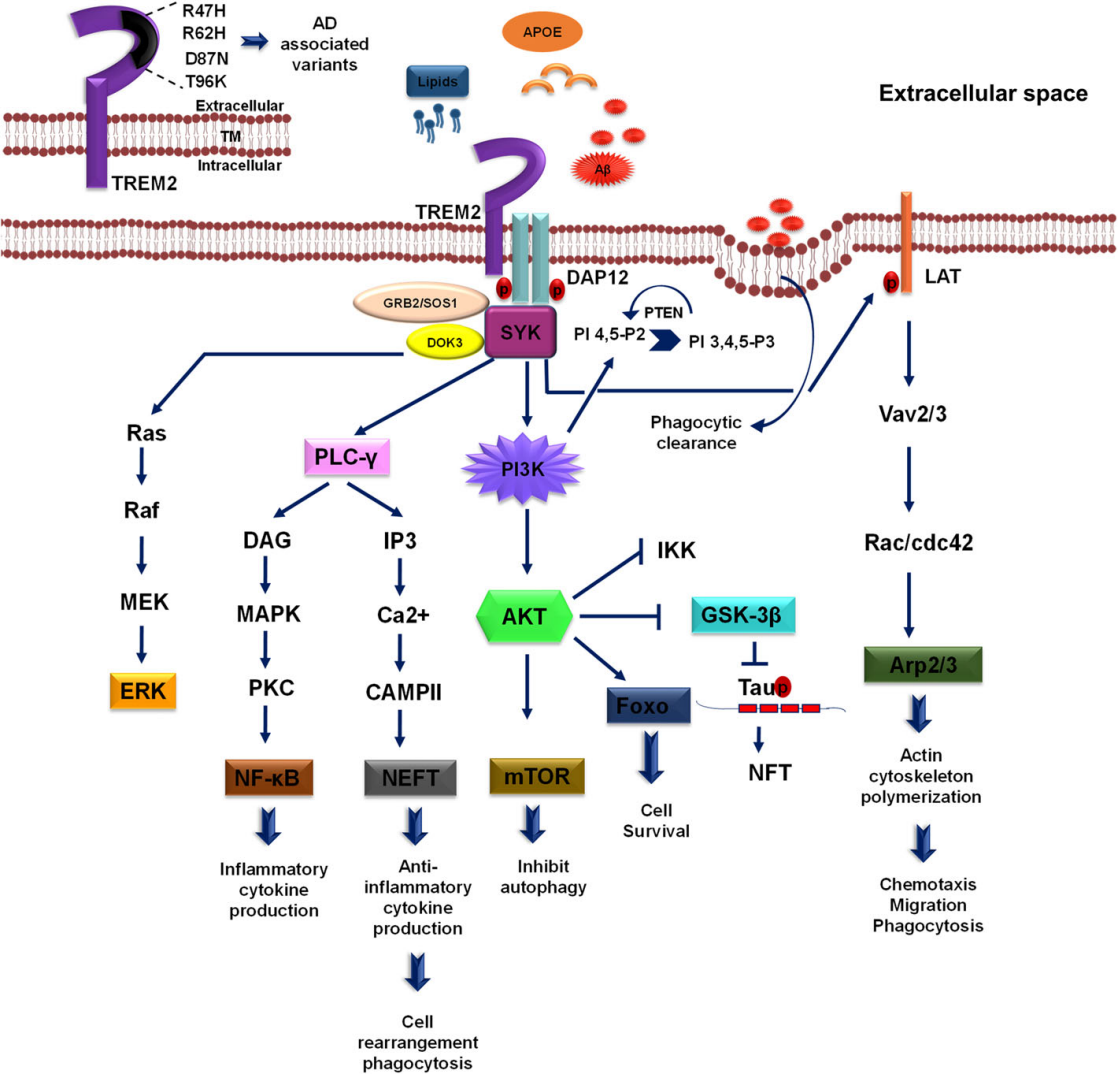
PI3K/Akt signaling-mediated by TREM2. DAP12-mediated TREM2 signaling have been discussed in the figure. The possible ligands with respect to Alzheimer’s disease for TREM2 are lipids, APOE as well as amyloid-β. In the physiological condition, upon tyrosine phosphorylation of DAP12 ITAM leads to recruitment of Syk kinase along with the GRB/SOS1 and DOK3, which activates downstream signaling pathways of ERK, PLC-γ, PI3K/Akt as well as Vav. The activation of PLC-γ induces inflammatory cytokine response *via* DAG stimulation and anti-inflammatory cytokine response along with cell rearrangement and phagocytosis via elevating Ca^2+^ response in the cell through IP3 activation. The main survival pathway of cell PI3K/Akt primarily activates mTOR pathway and Foxo that inhibit autophagy and induce cell survival, inhibits GSK-3β that eventually reduce Tau phosphorylation in Alzheimer’s disease and suppress IKK, which is involved in the inflammatory response. PI3K activation increase formation of PI 3,4,5-P3, an important phosphoinositide in the process of phagocytosis and migration. Syk recruitment activates Vav2/3 molecule *via* LAT phosphorylation, which induces actin cytoskeleton polymerization important for chemotaxis, migration, and phagocytosis. TREM2 is one of the disease-linked genes in Alzheimer’s disease, the AD-related variant generally present in Ig-like extracellular domain of the receptor, which includes R47H, R62H, D87N, T96K

Triggering receptor expressed on myeloid cells 2 (TREM2) that belongs to immunoglobulin superfamily is an innate immune receptor, which is exclusively expressed in myeloid cells such as immature dendritic cells, tissue macrophages, microglia and osteoclasts. The structure comprises of extracellular single v-type immunoglobulin domain, short ectodomain, transmembrane region and short cytoplasmic tail [76, 77]. TREM2 contributes to various cellular processes such as regulation of inflammatory cytokine production, phagocytosis, migration, proliferation, etc. [78]. With recent studies, it has been shown that TREM2 is associated with the adaptor protein, DNAX activating protein 12 (DAP12) and DAP10. Upon ligand binding, these adaptor proteins undergo phosphorylation to activate downstream signaling cascades. DAP12 is known to mediate activation of spleen tyrosine kinase (Syk), and DAP10 propagates the signal by recruiting PI3K. TREM2 binds to DAP12 or DAP10 and forms heterodimers [79]. DAP10 is critical in the activation of Akt and extracellular signal-regulated kinase (ERK), whereas DAP12 is essential for Ca^2+^ mobilization [79, 80]. DAP12 is a type I transmembrane adaptor protein, which shares immunoreceptor tyrosine-based activation motifs (ITAM) in cytoplasmic domain, charged acidic transmembrane region, and also bears capacity to recruit src-homology domain-2 (SH2) on tyrosine phosphorylation. DAP12 is homodimeric, where cysteine residues (Cys33 and Cys35) at extracellular domain allow homodimer formation. Phosphorylation of tyrosine residues on ITAM by Src protein tyrosine kinase provides docking site for SH2 domain such as spleen tyrosine kinase (Syk) [81–84]. TREM2 is activated through a wide variety of ligands such as bacterial products, lipoproteins (LDL), phospholipids (phosphatidylserine, cardiolipin), glycolipids, lipopolysaccharides (LPS), APOE and Aβ [85, 86]. Similarly, clearance of extracellular bacteria carried out through pattern recognition receptors (PRR), TREM2 and its adaptor protein DAP12 implement binding and uptake of a varied class of gram-negative and positive bacteria [87]. The activation of TREM2/DAP12 successively recruits PI3K after interaction between DAP12 and p85 subunit of PI3K, suggesting its involvement in TREM2 signaling [88]. PI3K targets Akt activation that further phosphorylates downstream targets, which include IKK, p21, caspase 9 and mTOR, depending upon the response. TREM2-mediated activation of PI3K/Akt is involved in reactive oxygen species (ROS) generation necessary to kill the internalized pathogens in macrophages [89]. The signaling pathway controls microglial survival by proliferation and reduced apoptosis by stabilizing β-catenin *via* Akt/GSK3β mechanism, which initiates the expression of responsible genes Cyclin D1, c-Myc and Bcl-2 [90]. TREM2 upregulates expression of CCR7, promotes chemokine-mediated response and enhances phagocytosis *via* ERK-dependent pathway. The signaling pathway is a legitimate mediator to balance pro-inflammatory and anti-inflammatory activity by microglia [78]. TREM2 signaling is directed towards anti-inflammatory response, which profusely antagonizes TLR-4-mediated inflammatory response by modulating JNK and NF-κB pathway [91]. TREM2 and DAP12 overexpression is observed to suppress the IL-6 and IL-1β response, anticipate the role of TREM2 signaling in the suppression of LPS-mediated inflammatory response [78, 92]. Further, the capacity of TREM2-DAP12 expression to influence the phagocytosis has been linked with enrichment of microglia-mediated cytoskeleton rearrangements. The pathway follows tyrosine phosphorylation of DAP12, Syk, Rac1 and cdc42. DAP12 signal mediates through Vav guanine nucleotide exchange factor family protein Vav2 and Vav3, which have the potential of activating Rho, Rac and cdc42. The pathway to exerbate phagocytosis *via* TREM2-DAP12 includes Src-Syk-Vav2/3-Rac1/cdc42-Arp2/3, which initiates necessary cytoskeleton rearrangement for phagocytosis [93, 94]. Hence, TREM2 mediated PI3K/Akt activation is a key pathway to control inflammatory response, apoptosis, survival pathway, and phagocytosis by microglia [81, 95] (Fig. 2).

### TREM2-mediated signaling in Alzheimer’s disease

TREM2 is constitutively expressed in microglia which are surrounded by amyloid plaque for the phagocytic clearance in AD. Amyloid-β oligomers found to bind with TREM-2 with nanomolar affinity, however AD-related mutations in TREM2, reduce its affinity towards binding and clearance of Aβ oligomers [96]. Aβ oligomers induce TREM2-mediated activation of microglial signaling pathways and clustering of microglia around the plaque regions in brain [97]. The dysfunction of TREM2-DAP12 axis is one of the major reason for impaired clearance of plaques, elevated pro-inflammatory activity by microglia in AD [98]. The heterozygous diseased mutants R47H and R62H of TREM2 recently has been identified as a risk factor for AD [99, 100] (Fig. 2). The TREM2 diseased variant drastically impedes the phagocytosis and the capacity to avert NF-κB activation in microglia. Aβ oligomers have a high affinity towards TREM2, which activate nuclear factor for activated T cells (NFAT). However, in TREM2 AD variant, a partial loss of TREM2 functioning has been observed [101]. TREM2 deficiency have been observed to reduce microglial viability, proliferation, microgliosis and decrease stability of β-catenin, an important factor of canonical Wnt signaling pathways. TREM-2-mediated stability of β-catenin is maintained by inhibiting its degradation *via* Akt/GSK-3β pathway. TREM2 knockdown decreases the level of AKT^S473^ and GSK-3β^S9^, which indicates that TREM-2 mediated stabilization of β-catenin is *via* AKT/GSK-3β signaling pathway [90, 102]. Disrupted PI3K/Akt signaling affects downstream factors as well, such as mTOR, a regulator of autophagy. Decreased activation of mTOR elevates excessive accumulation of autophagosomes, metabolic impairment and inability of microglia to clear Aβ plaques [103]. The recent finding of disease-associated microglia (DAM) phenotype importantly stresses upon the TREM2-PI3K-Akt signaling axis for its development [104]. TREM2 mutation dramatically hampers TREM-2–mediated phagocytosis, which is mediated by SYK/PI3K/Akt/PLCγ signaling pathway. The mutation also decreases the inhibition of NF-κB-mediated inflammatory response [95]. Hence, TREM2 deliberately reduce neuroinflammation and improves neuronal functions *via* PI3K/Akt-mediated signaling cascade [105]. Effect of TREM2 deficiency also elevates Tau pathology, but the mechanism is still needs to be addressed [106] (Fig. 2).

## Role of GPCR regulating/ interacting proteins in Alzheimer’s disease

### Small GTPases

Small GTPases belongs to the group of G proteins that are capable of hydrolysing GTPs to form GDPs [48]. Small GTPases constitute more than 100 different proteins and are broadly classified into five families, i.e. Ras family of GTPases, Rab family of GTPases, Rho family of GTPases, Ran GTPases and Arf GTPases [48]. GTPases are regulated by three main regulatory proteins, Guanine nucleotide exchange factors (GEFs), GTPase activating proteins (GAPs) and Guanosine nucleotide dissociation inhibitors (GDIs) [107–109]. GEFs are involved in the activation of GTPases by catalyzing the exchange of GTPs for GDPs, whereas GAPs are involved in the inactivation of small GTPases by promoting GTP hydrolysis [107, 108]. GDIs maintain the inactive form of GTPases by preventing its membrane localization [109]. GTPases respond to various extracellular stimuli and are involved in cell survival via various downstream signaling cascades that regulate gene expression [48]. Rho (RhoA, Rac1 and Cdc42) signaling cascades play a crucial role in actin and tubulin dynamics of neuronal cells [110, 111]. Several membrane receptors such as G_12/13_-protein coupled GPCRs, tyrosine kinase receptors, integrin receptors are involved in the activation of Rho signaling pathway [112–114]. The receptors are mainly involved in the activation of different Rho-GEFs: p115-RhoGEF, PDZ-RhoGEF, leukaemia-associated RhoGEF and lymphoid blast crisis-RhoGEF, [115, 116] which further activates Rho-GTPases by the exchange of GTP for a GDP molecule. In the human brain, Rho GTPases are involved in a wide variety of functions such as learning and memory formation (by maturation of dendritic spines), neuronal survival, cell migration via actin cytoskeleton dynamics, microtubule stabilization, etc., [111, 117–119]. Rab-GTPases are the key regulators of intracellular membrane trafficking and are involved in several vital functions of CNS development [120–123]. Arf GTPases are involved in the vesicular transport similar to Rab proteins [124]. Ran GTPases regulate the nucleo-cytoplasmic transport of proteins and RNA in the cell [125]. GTPases are involved in a wide variety of functions ranging from vesicular trafficking, membrane transport, actin networking, *etc*., In the human brain, small GTPases are involved in the regulation of various functions such as phagocytosis and vesicular trafficking by Rab and Arf family of GTPases in microglia; actin remodelling and microglial migration by Rho GTPases; maturation of dendritic spines and neuronal survival by Rho GTPases, *etc.* The signaling of these different GTPases and their pathological role in Alzheimer’s disease have been widely described.

### Rho family of GTPases in amyloid-β and Tau pathology

Rho family of GTPases are primarily involved in actin cytoskeleton remodelling and constitutes around 20 members that include RhoA, Rac1, Cdc42, etc., [117, 126]. Activation of Rho GTPases is GEF-mediated that are regulated by G_12/13_ proteins. G_12/13_ proteins are involved in the translocation of Rho-GEF from cytosolic region to the plasma membrane for its activation. Activated membrane-bound Rho-GEF in turn catalyzes the activation of Rho GTPases. Similarly, Rho-GTPases are also activated by different receptor tyrosine kinases and integrin receptors. 8 Rho-GTPases among 20 are known to be activated by RTKs [113]. Upon activation, each of these GTPases has a distinct set of effector kinases that are involved in different physiological functions of the cell. For example, RhoA effector kinases include Rho-associated protein kinases (ROCK1 and ROCK2), Citron, PKN/PRK1, etc. Cdc42 effector kinases include myotonic dystrophy kinase-related CDC42-binding kinase (MRCK), activated Cdc42 kinase (ACK), mixed lineage kinases (MLK), p-21 activated kinases (PAK), etc. Rac1 effector kinases include mixed lineage kinases (MLK) and p-21 activated kinases (PAK) which act as common effectors for Rac1 and Cdc42. In AD, synaptic degeneration and impaired actin cytoskeletal network has been observed which is mainly mediated by the pathological effects of these Rho GTPases [127–129].

#### RhoA GTPases

**Fig. 3:**
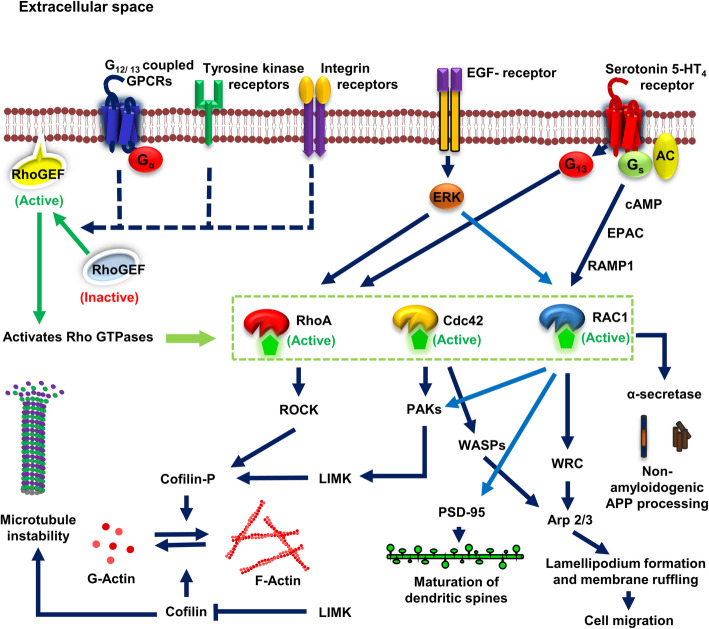
Physiological activation and signaling of Rho-GTPases. Several membrane receptors, such as G_12/13_ coupled GPCRs, Tyrosine kinase receptors, Integrin receptors, etc., are involved in the activation of Rho-GEFs. The active form of Rho-GEF binds to the membrane and are involved in activation of Rho-GTPases (mostly RhoA, Rac1 and Cdc42) through GTP catalysis. Epidermal growth factor receptor is involved in ERK-mediated phosphorylation of RhoA and Rac1 and its activation. Serotonin 5-HT4 receptor activates cAMP, EPAC and RAMP1 *via* G_s_ signaling and activates Rac1 signaling that is involved in the promotion of non-amyloidogenic APP processing, dendritic spine formation (PSD-95 expression) in mature neurons, lamellipodium formation and membrane ruffling for migration. Serotonin 5-HT4 receptor also activates RhoA *via* G_13_ proteins. Activated RHO-GTPases are involved in several vital functions such as cell migration, maturation of dendritic spines, neuritic outgrowth, learning, memory, *etc*., RhoA GTPase activates ROCK kinase that is involved in the phosphorylation of cofilin. Cofilin is involved in the formation of globular actin from filamentous actin (actin depolymerization), and this process is inhibited and reversed upon cofilin phosphorylation. Cofilin also binds tubulin and promotes microtubule instability. Mixed lineage kinases (MLK) and p-21 activated kinases (PAK) are the common effectors of Cdc42 and Rac1. PAKs are involved in the activation of LIM kinase, which also involved in the phosphorylation of cofilin. Wiskott–Aldrich syndrome protein (WASP) and WAVE regulatory complex (WRC), the downstream effectors of Cdc42 and Rac1 respectively activate Arp 2/3 complex which promotes membrane ruffling during migration

**Fig. 4:**
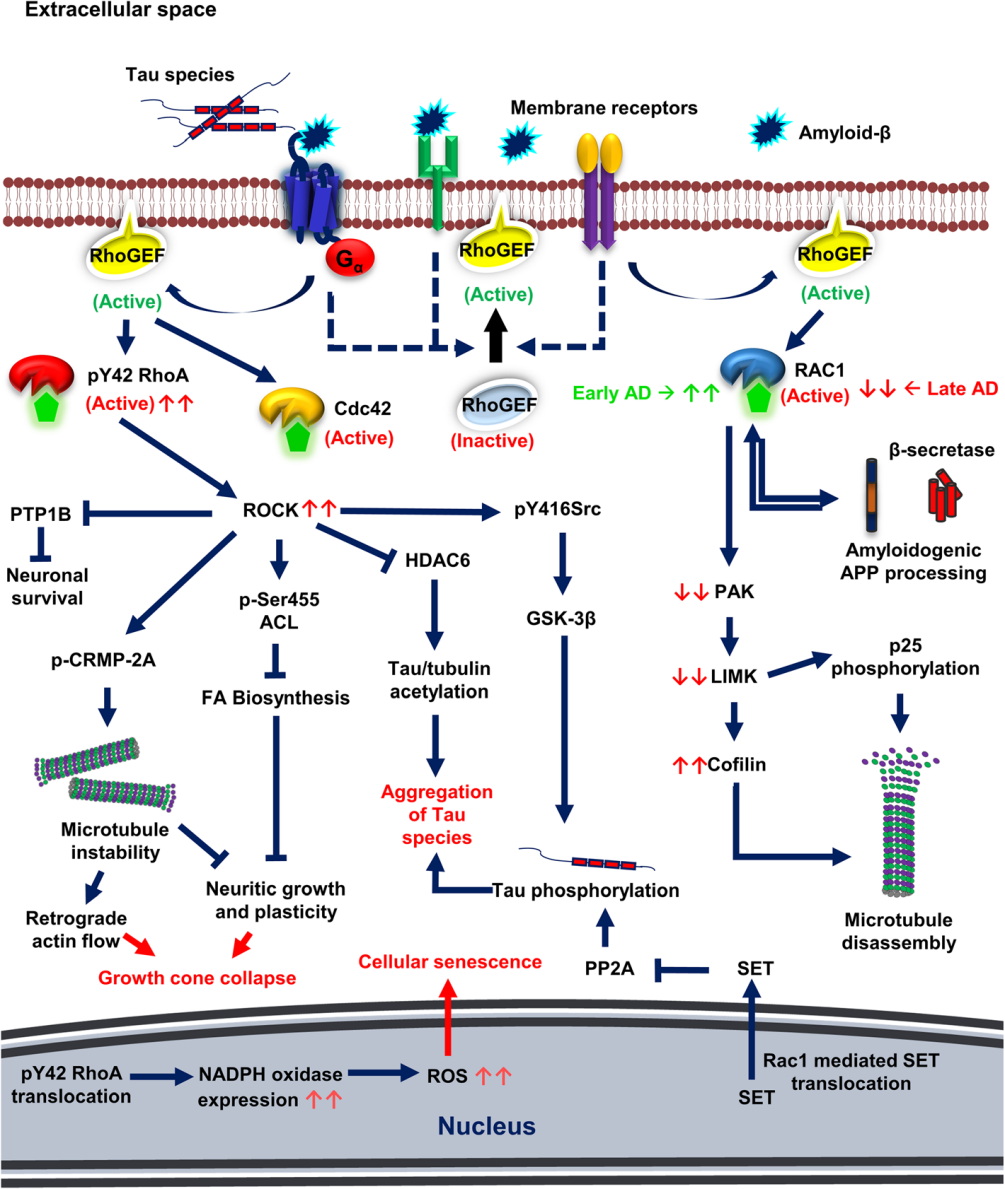
Pathological signaling of Rho GTPases in Alzheimer’s disease. In Alzheimer’s disease, the extracellular space is filled with senile plaques of amyloid-β and aggregated Tau species. These disordered proteins interact with several surface receptors, which on activation lead to pathological signaling of Rho-GTPases. Rho-GTPases are highly activated (phosphorylated on Y42) on amyloid-β exposure. Hyperactivation of Rho-GTPases leads to increased activation of ROCK kinases. ROCK kinase phosphorylates Src at Tyrosine 416 and activates to activate GSK-3β, an enzyme involved in Tau hyperphosphorylation. ROCK also inhibits HDAC6 activity which ultimately promotes Tau and tubulin acetylation. All these post-translational modifications of Tau lead to aggregation of Tau species. ROCK phosphorylates and inactivates CRMP-2A and ATP citrate lyase (p-Ser 455) (ACL). CRMP-2A are microtubule-associated proteins, and its inactivation led to microtubule instability and retrograde flow of actin filaments. Fatty acid biosynthesis is inhibited by ATP-citrate lyase phosphorylation which ultimately inhibits neurite growth, plasticity and leads to growth cone collapse in neuronal cells. Activated ROCK kinases also inhibit protein tyrosine phosphatase 1B and neuronal survival. Phosphorylated RhoA translocates to the nucleus and promotes the expression of NADPH oxidase, increases ROS levels and cellular senescence. Rac1 GTPase expression level varies at different stages and promotes amyloidogenic APP processing during the later stages of Alzheimer’s disease leading to more amyloid-β secretion and *vice versa.* At later stages, Rac1 expression level is reduced, and subsequent effectors PAK and LIMK activities are also downregulated. This condition leads to the imbalance in cofilin (active) and its phosphorylated form (inactive). Excessive cofilin activity causes actin depolymerization and microtubule instability. Under AD conditions, Rac1 also promotes translocation of SET proteins from the nucleus to the cytoplasm that acts as phosphatase inhibitors and ultimately promotes phosphorylation and aggregation of Tau species

RhoA GTPases are majorly associated with synapses and dendritic microtubules [130]. G_12/13_ coupled receptors are involved in the activation of RhoA GTPases mediated by Rho-GEFs. Several G_12/13_-protein linked GPCRs, tyrosine kinase receptors, integrin receptors are involved in the activation of RhoA GTPases. One such receptor is serotonin 5-HT_4_ receptor which is reported to play a key role in learning, memory, behaviour and synaptic plasticity. Schill *et al.* 2020 reported that serotonin 5-HT_4_ receptor activates RhoA-mediated signaling via G_13_ proteins that leads to the phosphorylation of cofilin by ROCK kinases [131]. Cofilin is a key molecule that has a significant role in stabilization and reorganization of the actin cytoskeleton. RhoA activation by 5-HT_4_ receptor agonist in primary cultures of hippocampal neurons promoted the formation of filamentous actin and maturation of dendritic spines [131] (Fig. 3). In neurodegenerative diseases such as AD, RhoA GTPases are playing a crucial role in promoting the progression of the disease. The activity of p-Tyr42 Rho was observed to be significantly higher in AD mice models and human brain of AD patients [132]. Amyloid-β disrupts microtubule stability and actin polymerization *via* activation of RhoA GTPases [132, 133]. Amyloid-β promoted GTP-bound RhoA in human SH-SY5Y cells and the cellular localization of RhoA was increased in dystrophic neurites of APP mice models [130, 133]. Amyloid-β exposure on SH-SY5Y cells also showed increased expression levels of collapsin response mediator protein-2A (CRMP-2A) and its phosphorylated form (phospho-threonine) [133]. CRMPs are a class of microtubule-associated proteins that are involved in a wide variety of neuronal functions such as neuronal development, dendritic formation, axonal guidance and also in cell migration [134]. Phosphorylation of CRMP-2A mediated by amyloid-β/RhoA-GTP in Tg2576 mice models showed reduced levels of tubulin binding to CRMP-2A, which could prevent neuritic growth and plasticity [133]. Protein tyrosine phosphatases 1B (PTP1B) which are involved in neuronal survival are also inhibited by amyloid-β activated RhoA-GTPases [135]. Histone deacetyl transferace-6 (HDAC6) is involved in the deacetylation of tubulin and Tau [136, 137]. P-Tyr42 RhoA activates ROCK, which in turn inhibits HDAC6 activation and promotes tubulin and Tau acetylation, which would lead to microtubule instability and Tau aggregation respectively [132]. Immunohistological analysis revealed that RhoA colocalized with hyperphosphorylated Tau in human AD cortex and hippocampus [130]. Cap *et al*. 2020 demonstrated the role of RhoA/ROCK signaling towards the phosphorylation of Tau, expression of NADPH oxidase and subsequent generation of ROS [138]. Amyloid-β exposure activated RhoA (p-Tyr42) which in turn activates GSK-3β, a serine-threonine kinase majorly involved in hyperphosphorylation of Tau species [138]. At higher concentrations of amyloid-β, nuclear translocation of P-Tyr42 RhoA was observed and regulated the expression of NAD kinase by binding to NADK promoter which was similar to ROS exposure [138]. Amyloid-β mediated Rho/ROCK signaling also mediates retrograde flow of actin cytoskeleton that leads to growth cone collapse and microtubule destabilization [132] (Fig. 4).

#### Rac1 and Cdc42 GTPases

Rac GTPases (Rac1 and Rac3) are mainly involved in the formation of dendritic spines and play a vital role in learning, memory and synaptic plasticity [139]. Rac1 and Cdc42 are involved in the polymerization of actin cytoskeletal network, formation of lamellipodia structures and membrane ruffling and play a significant role in cell migration [118, 140]. Also, Rac1 plays an essential role in axonal growth, guidance and promotes neuronal survival in the central and peripheral nervous system [119]. p21-activated kinases (PAKs) are serine/threonine kinases that are direct effectors of Rac1/Cdc42-GTPases, which are majorly involved in actin remodelling [141]. Phosphorylation of regulatory light chains (RLC) on serine19 by PAKs leads to actin network stabilization and dendritic spine morphogenesis [142]. LIM kinase (LIMK), a serine-threonine kinase is another PAK effector that is involved in the promotion of filamentous actin and microtubule disassembly [143]. Phosphorylation of cofilin by LIMK inhibits its activity which leads to actin polymerization [143]. LIMK also inhibits p25 activity (promotes tubulin polymerization) by phosphorylation, thus leading to microtubule disassembly [143, 144] (Fig. 3). In AD brains, PAKs levels and activity are depleted due to amyloid-β and hence, active levels of cofilin increases, which ultimately leads to microtubule instability [145, 146]. It is also reported that activated cofilin directly binds tubulin, displaces Tau from tubulin/microtubules, and inhibits Tau-induced microtubule assembly [145]. Rac1 GTPases are reported to be involved in the non-amyloidogenic APP processing through serotonin 5-HT4 receptor activation followed by cAMP/Epac/Rap1/Rac1 signaling pathway [147, 148]. Both Rac1 and Cdc42 are involved in the negative regulation of RhoA by activating Rho-GAPs [149, 150]. The levels of Rac1 has been significantly altered in the frontal cortex and plasma of AD patients at different stages of disease progression when compared with age-matched controls [151]. Under pathological conditions, Rac1 GTPases are involved in amyloidogenic pathway of APP processing and promotes the formation of amyloid-β peptides [148, 151]. The Rac1-specific inhibitor NSC23766, decreased the levels of Rac1 and APP in a concentration-dependent manner [148]. Rac1 peptide treatment in primary cortical neurons and human SH-SY5Y neuroblastoma cell lines enhanced the immunoreactivity of amyloid-β peptides and/or its precursor, APP [151]. However, controversial results were observed during the later stages of disease progression. Rac1 level decreased in 7 months old 3xTg-AD mice models and Rac1 administration enhanced the expression of the post-synaptic marker, PSD-95Rac1, rescues spine loss and ameliorates synaptic abnormalities [151]. Rac1 GTPases also promotes phosphorylation of Tau protein by enhancing the translocation of nuclear oncoprotein, SET to the cytosol that acts as a protein phosphatase 2A (PP2A) inhibitor [151] (Fig. 4).

### Rab family of GTPases

Ras-like GTPases in brain (Rab) are the family of small GTPases with 61 members and are involved in regulating intracellular vesicular transport that includes both endocytosis and exocytosis [152]. In the brain, Rab GTPases are involved in several vital functions such as CNS development, polarized neurite growth, endocytosis and axonal retrograde transport, synaptic vesicle exocytosis, etc., (briefly reviewed by Ling NG *et al*. 2008) [120]. Several Rab GTPases such as Rab5, Rab7A, Rab10, Rab11A are reported to be associated with AD [153–155]. The levels of Rab5 and Rab7 were significantly upregulated in the frontal cortex and hippocampus of AD brain [156]. Rab5 GTPases are involved in trafficking and fusion of early endosomes and the Rab5 positive early endosomes are found enlarged with increased endocytosis under AD conditions [156–158]. It is also reported that Rab5-mediated APP processing led to apoptosis in AD neurons [158]. Rab11 GTPases are colocalized with BACE-1 during the late onset of AD that are involved in axonal sorting of BACE-1 [154, 159]. Rab7 GTPases are colocalized with BACE-1, the enzyme responsible for amyloidogenic APP processing and synthesis of amyloid-β peptides [72]. Rab7A is involved in multiple roles that include regulation of late endosomes and lysosomal degradation pathway [160]. In AD brain, Rab7A level is elevated and is reported that it regulates exosome-mediated Tau secretion [161]. Rab7A deletion on primary cortical neuronal cells and HeLa cells overexpressing Tau downregulated Tau secretion which clearly indicated the pathological role of Rab7A in Alzheimer’s disease and Tauopathy [161, 162].

### GPCR-mediated lysophosphatidic acid signaling

**Fig. 5:**
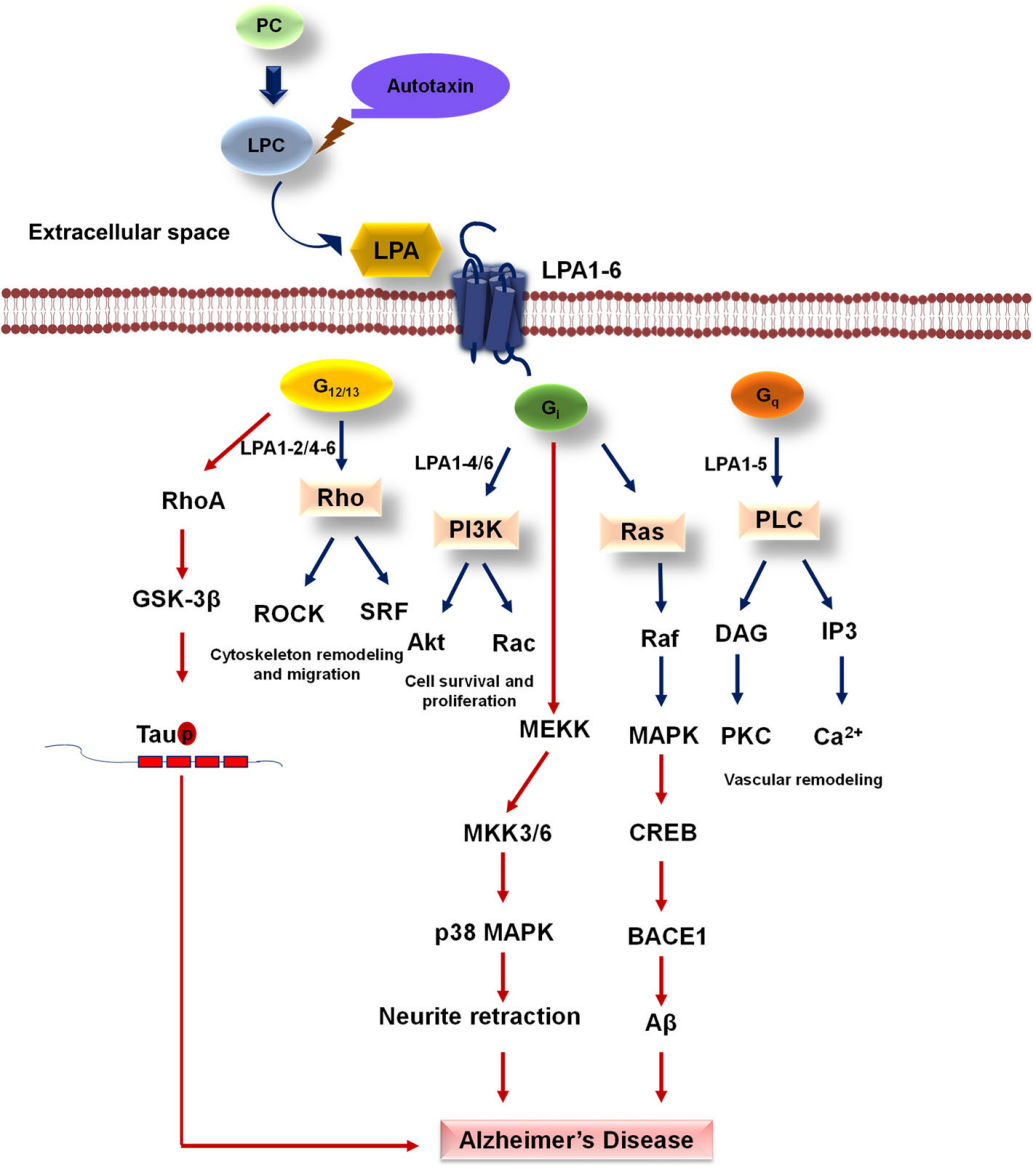
GPCR in Lysophosphatidic acid cycle. LPA production through LPC *via* autotaxin enzyme exerts its function through cell surface GPCR mainly G12/13, Gi/o, and Gαq protein-mediated signals. Amongst six different LPA, LPA1-2/4-6 exert their function through Gα_12/13_ and follows Rho-mediated cytoskeleton remodeling which assists cellular migration. LPA1-4/6 exert their function through Gi/o, which is responsible for activating PI3K and Ras-mediated MAPK signaling for cell survival and proliferation. Gαq protein-mediated signal activate the PLC pathway results in vascular remodeling on stimulation by LPA1-5. Defect in signaling through Gα_12/13_ in AD abnormally increases active GSK-3β, which increases phospho-Tau. Gi leads to dysregulation of BACE1, which impose abnormal Aβ and neurite retraction via p38 MAPK kinases

Lysophospholipids are the part of membrane phospholipid that comprises of a polar head group followed by a hydrophobic carbon chain. It comes under lyso-type glycerolipid having glycerol backbone with ester-linked fatty acids chain (acyl chain at position 1 or 2) and a phosphate group [163]. In humans, 16:0 LPA, 18:1 LPA, 18:2 LPA are commonly observed that are majorly expressed in astrocytes, microglia, neural progenitor cells and oligodendrocytes in the nervous system [164]. There are four pathways of LPA formation among which autotaxin (ATX) is majorly involved in cellular signaling cascades. Autotaxin is a pyrophosphatase/phosphodiesterase enzyme (ENPP), which hydrolyses phosphodiester bond of lysophospholipids, nucleoside triphosphates, and choline phosphate esters [165]. ATX importantly converts lysophospholipids into lysophosphatidic acid (LPA) for signaling in the cell [166]. ATX mediates cleavage of lysophosphatidylcholine (LPC) into LPA and choline and similarly, lysophosphatidylethanolamine, and lysophosphatidylserine into LPA and respective secondary group for the signaling pathway. LPA is also produced from phosphatidic acid (PA) *via* enzyme phospholipase A1α or β [167]. LPA-mediated physiological effects include nervous system development, immune system function, inflammation, and wound healing, which is executed by currently known six LPA receptors LPAR1-LPAR6 [168, 169]. LPA formed extracellularly act as a ligand for cognate GPCRs to activate signaling cascade, and the signal proceeds *via* Gα subunits such as G_12/13_, G_q/11_, G_i/o_, and G_s_ [170] (Fig. 5).

Neuronal progenitor cells (NPC) are the preliminary cells that initiate neurogenesis by differentiating into neurons and other glial cells as restricted cell types. LAP mediate neurogenesis of NPC *via* LPA1, LPA2, LPA4 receptors [171]. LPA1 plays an important role in modulating neurogenesis, i.e., the process of differentiation is driven by LPA1 and partly by LPA2. LPA exerbate neurotransmitter like stimuli of ionic conductance hence arbitrate cortical development [172, 173]. LPA1 function through G_i/o_ protein-mediated pathway, which implement Rho-mediated actin rearrangement, microtubule formation important for cell morphology and motility. LPA1 also initiates phospholipase C (PLC) activation *via* pertussis toxin (PTX)-insensitive pathway mediated by G_q_ proteins, making LPAR1 a multifunctional LPA receptor [174]. LPA1 especially induces synaptic plasticity that includes protein kinase c (PKC) activation, with Rho and Rac-dependent actin-based changes [175, 176]. Similarly, LPA2 also functions through G_i_ and G_q_ mediated pathways, which activates PLC, and increases intracellular Ca^2+^ and inositol phosphate production [177]. Extension and branching neurites are important for the formation of neuronal network, which requires actin rearrangements. The neurite retraction and growth cone collapse are induced by all LPA receptors except LPA3. One anticipated function of LPA3 is to increase intracellular Ca^2+^ levels, however, it induces axonal branching and neurite branching through G_q_ and the Rho family GTPase 2 (Rnd2) family [178, 179]. LPA4 receptor is linked with G_q_, G_12/13_ and to some extent with G_s_ protein. LPA4 reduces the LPA driven cell motility and invasion by negatively modulating PI3K pathways, Rho-dependent migration. It also enhances cell adhesion and aggregation [180]. Similarly, LPA5 is also an anti-migratory receptor, which signals through PKA-CAMP pathway and also responsible for reduced PIP3 levels at the plasma membrane that supports reduced migration [181]. However, LPA6 is recently characterized, but the mutation of this receptor leads to hair loss in humans [182]. Its function related to CNS still needs to addressed.

In AD, amyloidogenic pathway sequentially functions through β-secretase and γ-secretase that leads to the production of toxic Aβ peptide. β-secretase activity, also known as β-site APP-cleaving enzyme 1 (BACE1), is the rate limiting step in production of Aβ [182]. Additionally, vascular factors such as oxidized low-density lipoprotein (oxLDL) have been reported in AD pathogenesis. Human cerebrospinal fluid (CSF) consists of a high level of lipoprotein in AD, the occurrence of levels of Aβ and oxLDL indicated a significant role of oxLDL in Aβ production [183]. LPA is a major bioactive component of oxLDL, which are known to disrupt blood-brain barrier (BBB) and imparts various pathologies related to AD [184]. BACE expression increases by LPA *via* cAMP response element binding protein (CREB) mediated mechanism. LPA increases the binding affinity of CREB to CRE site of the BACE1 promoter sequence. Hence there is upregulation of BACE1 upon LPA response. The signaling pathway involves phosphorylation of PKCδ, MEK, MAPK and p90RSK, which mediate CREB phosphorylation leading to upregulation of BACE1 [166]. LPA induce Tau phosphorylation and neurite retraction, the major target of LPA to cause Tau phosphorylation is glycogen synthase kinase- 3β (GSK-3β) [185]. Increased activity of GSK-3β in response to LPA is carried out through G_α12/13_-mediated Rho/ROCK pathway. Rho-GTPase has been observed to cause Tau pathology, where Rho/ROCK pathway phosphorylates Tau at various epitopes such as Thr245 and Ser409 [186]. LPA-induced neurite retraction is carried out after phosphorylation of Tau by GSK-3β, PKA, and additionally with activation of p38 MAPK [187] (Fig. 5).

### Other GPCR interacting proteins in amyloid-β and Tau pathology

G-protein coupled receptor kinases (GRKs) and β-arrestins are the first identified GPCR/ G-protein regulating proteins that are involved in receptor desensitization and endocytosis [188]. GRKs are involved in phosphorylation of receptor upon ligand activation [189–191]. Arrestin binds to phosphorylated GPCRs in order to prevent its coupling to G-proteins, ultimately leading to receptor desensitization and endocytosis [192, 193]. β-arrestin 1 and 2 are majorly involved in receptor desensitization, followed by internalization which mediates several G-protein independent signaling pathways that includes activation of cellular kinases, transactivation, gene expression, etc., [194, 195]. The internalized GPCRs further undergoes endosomal or lysosomal sorting for dephosphorylation/resensitization or lysosomal degradation respectively [196, 197]. The expression level of β-arrestin varies during the progression of AD. β-arrestin 2 expression was observed to be elevated in AD patients [198]. Several GPCRs such as GPR3, β_2_ adrenergic receptor and δ-opioid receptors are involved in G-protein independent regulation of amyloid-β synthesis *via* γ-secretase activity [72, 199, 200]. Later, Thathiah *et al*. 2013 demonstrated that these receptors are involved in regulation of amyloid-β production and γ-secretase activity by directly activating β-arrestin 2 [198]. Gene knock-out studies for β-arrestin 2 failed to produce amyloid-β, which indicated β-arrestin-mediated GPCR signaling [198]. β-arrestin 1 is also involved in elevated amyloid-β synthesis mediated by γ-secretase activity [201]. Both β-arrestin 1 and 2 are reported to bind directly to anterior pharynx-defective 1α (APH-1α) subunit of γ-secretase for its activation [198, 201]. There are other GPCR regulating proteins that are involved in the pathogenic progression of Alzheimer’s disease. Ras homolog enriched in brain (Rheb) are another group of small GTPases that are highly expressed in frontal cortex and hippocampus [202]. Rheb GTPases are the key regulators of mTOR signaling pathways and are involved in the regulation of BACE1 activity and degradation [203, 204]. Overexpression of Rheb in cultured neuronal cells attenuated BACE1 and Aβ levels in mTOR-independent pathway [204]. In AD brains, the levels of Rheb is downregulated, which lead to the enhanced activation of BACE1 and Aβ production [204]. GPCR-associated sorting proteins (GASPs) are the group of sorting proteins that promotes lysosomal degradation of internalized receptors [205]. p60TRP (transcription regulator protein) is a GASP that are involved in neuronal survival and rescues cells from death [206, 207]. Overexpression of p60TRP in P-12 cells promoted dephosphorylation of APP and downregulated BACE1 activity [207].

## Conclusion

Approximately, 481 drug molecules (~34%) are approved by the FDA act that targets GPCRs. 320 drugs are under clinical trials among which ~36% could be potentially novel GPCR targets. Among CNS diseases, around 130 drugs (27%) are FDA approved that are potential GPCR targets [208]. The mis-regulation of signaling pathways in Alzheimer’s disease have serious impact on amyloid-β secretion, Tau phosphorylation, neurotransmitter signaling, glial function which eventually ameliorate the disease condition. The cell surface receptors, GPCR and the subsequent GPCR related signaling molecule are involved in regulation of BACE1, γ-secretase and eventually affect Tau phosphorylation in Alzheimer’s disease. Several drug molecules have been developed in the field of Alzheimer’s disease targeting a wide variety of receptors such as G-protein coupled receptors, kinases/ enzymes, ionotropic channels, hormone receptors, etc., that largely failed during clinical trials [209]. Allosteric modulators could be an alternative and effective therapeutic agent for Alzheimer’s disease. For example, positive allosteric modulators (PAM) of M1 muscarinic acetyl choline receptor are proved to be effective in AD therapy [210]. Hence, understanding the role of G-protein coupled receptor signaling pathways and the molecules involved in the pathway is necessary to design a particular drug target to treat the disease. With the evolved knowledge regarding cell surface receptors, GPCR as well as interacting GTPase might provide alternative strategies to modulate the AD pathogenesis.

## Data Availability

Not applicable.

## Abbreviations

Aβ: Amyloid-β
AD: Alzheimer’s disease
ACK: Activated Cdc42 kinase
APH-1α: Anterior pharynx-defective 1α
APP: Amyloid precursor protein
Arf: ADP-ribosylation factor
ATX: Autotaxin
BACE1: β-site APP-cleaving enzyme 1
BBB: Blood-brain barrier
CNS: Central nervous system
CREB: CAMP response element binding protein
CRMP-2A: Collapsin response mediator protein-2A
CSF: Cerebrospinal fluid
DHA: Docosahexaenoic acid
DOR: Delta-opioid receptor
FSH: Follicle stimulating hormone
GAP: GTPase activating protein
GASP: GPCR-associated sorting protein
GDI: Guanosine nucleotide dissociation inhibitor
GDP: Guanosine diphosphate
GEF: Guanine nucleotide exchange factor
GPCR: G-protein coupled receptor
GSK3β: Glycogen synthase kinase 3β
GTP: Guanosine triphosphate
HDAC6: Histone deacetyl transferase 6
ITAM: Immunoreceptor tyrosine-based activation motifs
LIMK: LIM kinase
LPA: Lysophosphatidic acid
LPC: Lysophosphatidylcholine
LPS: Lipopolysaccharides
MAPK: Mitogen activated protein-kinases
MLK: Mixed lineage kinase
MRCK: Myotonic dystrophy kinase-related CDC42-binding kinase
NADK: Nicotinamide adenine dinucleotide kinase
oxLDL: Oxidized low-density lipoprotein
PAK: p-21 activated kinase
PDK: Phosphoinositide-dependent kinase
PIP3: Phosphatidylinositol 3,4,5- triphosphate
PKC: Protein kinase C
PKN/PRK: Protein kinase-C related kinase
PP2A: Protein phosphatase 2A
PUFA: Polyunsaturated fatty acids
PIP2: Phosphatidylinositol 4,5 bisphosphate
PTEN: Phosphatase and tensin homologue
PTP1B: Protein tyrosine phosphatase 1B
Rab: Ras-like proteins in brain
Ras: Ras Sarcoma oncoproteins
Ran: Ras-like nuclear proteins
Rheb: Ras homolog enriched in brain
Rho: Ras homologous proteins
RLC: Regulatory light chain
ROCK: Rho-associated protein kinase
ROS: Reactive oxygen species
RTK: Receptor tyrosine kinase
SHIP: SH-domain-containing inositol polyphosphate 5-phosphatase
Syk: Spleen tyrosine kinase
TREM2: Triggering receptor expressed on myeloid cells 2
WASP: Wiskott–Aldrich syndrome protein
WRC: WAVE regulatory complex
